# C9ORF72 Is Pivotal to Maintain a Proper Protein Homeostasis in Mouse Skeletal Muscle

**DOI:** 10.3390/cells14221765

**Published:** 2025-11-11

**Authors:** Francesca Sironi, Paola Parlanti, Cassandra Margotta, Jessica Cassarà, Valentina Bonetto, Caterina Bendotti, Massimo Tortarolo, Valentina Cappello

**Affiliations:** 1 Research Center for ALS, Department of Neuroscience, Istituto di Ricerche Farmacologiche Mario Negri IRCCS, 20156 Milan, Italy; francesca.sironi3@gmail.com (F.S.); cassandra.margotta@marionegri.it (C.M.); cassara.jessica@gmail.com (J.C.); valentina.bonetto@marionegri.it (V.B.); massimo.tortarolo@marionegri.it (M.T.); 2 Center for Materials Interfaces, Istituto Italiano di Tecnologia, Electron Crystallography, 56025 Pontedera, Italy; paola.parlanti@iit.it (P.P.); valentina.cappello@iit.it (V.C.)

**Keywords:** amyotrophic lateral sclerosis, skeletal muscle, atrogenes, autophagy, mitochondria, mitophagy

## Abstract

**Highlights:**

**What are the main findings?**

- Deletion of C9ORF72 in mice results in a significant reduction in large muscle fibers, accompanied by increased proteasomal and autophagic activity.
- Loss of C9orf72 in skeletal muscle disrupts mitophagy and leads to ultrastructural mitochondrial abnormalities.

**What are the implications of the main findings?**

- C9ORF72 plays a critical role in maintaining mitochondrial integrity and proteostatic balance in skeletal muscle.
- Although C9ORF72 loss of function does not produce overt muscle weakness, it may render muscle fibers more vulnerable to degeneration in C9orf72-linked ALS.

**Abstract:**

The C9ORF72 gene mutation is a major cause of amyotrophic lateral sclerosis (ALS). Disease mechanisms involve both loss of C9ORF72 protein function and toxic effects from hexanucleotide repeat expansions. Although its role in neurons and the immune system is well studied, the impact of C9ORF72 deficiency on skeletal muscle is not yet well understood, despite muscle involvement being a key feature in ALS pathology linked to this mutation. This study examined skeletal muscle from C9ORF72 knockout mice and found a 19.5% reduction in large muscle fibers and altered fiber composition. Ultrastructural analysis revealed mitochondrial abnormalities, including smaller size, pale matrix, and disorganized cristae. Molecular assessments showed increased expression of Atrogin-1, indicating elevated proteasomal degradation, and markers of enhanced autophagy, such as elevated LC3BII/LC3BI ratio, Beclin-1, and reduced p62. Mitochondrial quality control was impaired, with a 3.6-fold increase in PINK1, upregulation of TOM20, reduced Parkin, and decreased PGC-1α, suggesting disrupted mitophagy and mitochondrial biogenesis. These changes led to the accumulation of damaged mitochondria. Overall, the study demonstrates that C9ORF72 is critical for maintaining muscle protein and mitochondrial homeostasis. While C9orf72-haploinsufficiency does not directly compromise muscle strength in mice, it may increase the vulnerability of skeletal muscle in C9ORF72-associated ALS.

## 1. Introduction

Amyotrophic lateral sclerosis (ALS) is a fatal motor neuron disease characterized by progressive skeletal muscle weakness and paralysis, leading to a premature death. Approximately 10% of ALS cases are familial, mostly characterized by autosomal dominant inheritance, while the remaining 90% are sporadic and of unknown etiology [1]. Mutations in the chromosome 9 open reading frame 72 (C9ORF72) gene are among the most common genetic causes of inherited ALS (40%) and frontotemporal dementia (FTD) (25%). These mutations are also significant in sporadic ALS (5–20%) and FTD (6%), explaining the overlap of the two conditions [2,3]. The high prevalence of C9ORF72-related ALS may be influenced by the presence of additional ALS-linked mutations [4].

The hallmark of the C9ORF72 mutation is the presence of an expanded G4C2 hexanucleotide repeat (HRE) in the first intron of the gene on chromosome 9p21 [5]. This abnormal repeat induces toxicity in neurons and other cells through three primary mechanisms: (1) toxic RNA gain-of-function as the long RNA fragments form foci that interfere with the functions of specific RNA-binding proteins; (2) toxic dipeptide repeat proteins (DPRs) produced by repeat-associated non-ATG (RAN) translation of mutant C9ORF72; (3) and loss of protein function due to the reduced expression of the C9orf72 transcripts and proteins [6]. HRE-mediated toxicity has been extensively studied in Drosophila [7,8], mice [9,10] and patients derived neurons [11,12], suggesting mechanisms such as perturbations in proteostasis [13], RNA metabolism [14], stress granule dynamics [15], lysosomal and nucleocytoplasmic transport systems [16]. However, the HRE can be pathogenic also through loss-of-function mechanisms by disrupting the expression of the C9orf72 gene being located in the promoter region. In fact, C9ORF72 expression is reduced in C9ALS/FTD patient tissues [2]. Interestingly, while ablation of the zebrafish and C. Elegans C9orf72 orthologs resulted in motor neuron degeneration [17,18], mice with deleted expression of C9orf72 failed to exhibit a neurodegenerative phenotype and motor impairment [19,20,21,22,23]. However, the elimination of mouse endogenous C9orf72 in various C9orf72 transgenic mice expressing HRE produced significant motor deficits [24,25], indicating a synergistic interaction between gain and loss of function mechanisms in ALS pathogenesis even if such interaction was not accompanied by the loss of ChAT positive spinal motor neuron or motor axons [25], suggesting a potential impact of the C9orf72 deficiency in skeletal muscle.

C9orf72 is a DENN (differentially expressed in normal cells and neoplasia)-like protein, which regulates small GTPases and autophagy by interacting with Rab family members and forming complexes with SMCR8 and WDR41 [26,27]. C9ORF72 regulates endosomal trafficking, lysosomal biogenesis, and autophagy in cell culture models. Its deficiency induced by siRNAS-inhibited endocytosis causes the accumulation of autophagosomes [28]. However, the loss of C9orf72 was also reported to upregulate the autophagy-inducing transcription factor known as transcription factor EB (TFEB) in both human cells and the mouse brain by inhibiting the mTOR signaling [29]. Thus, whether the reduced level of C9orf72 lead to impaired or enhanced autophagy and at which steps of the autophagic pathway remains controversial and appears to depend on the model system used [30].

C9ORF72 is also implicated in mitochondrial quality control, influencing oxidative phosphorylation, mitochondrial fission, and mitophagy, critical processes in cellular energy homeostasis [31].

While ALS is traditionally regarded as a motor neuron disease, growing evidence suggests that skeletal muscle dysfunction actively contributes to its pathogenesis [32]. Impaired proteostasis and autophagy–lysosome dysfunction are key mechanisms in ALS pathogenesis, not only in neurons but also in skeletal muscle [33,34]. Autophagy impairments, mitochondrial defects, and imbalanced proteostasis can lead to muscle fiber atrophy and metabolic dysfunction, even preceding overt motor symptoms [35].

The autophagy–lysosome system is responsible for the selective degradation of protein aggregates and organelles, such as the mitochondria (i.e., mitophagy). This process, together with mitochondrial biogenesis, is essential for maintaining healthy and functioning muscle fibers [36].

Recent studies have identified DPR-related pathology in skeletal muscle from ALS patients and animal models with C9ORF72 mutations [37,38]. However, whether skeletal muscle experiences haploinsufficiency of C9ORF72 protein remains largely unexplored. Only a short report showed a loss of C9ORF72 expression in the gastrocnemius muscle biopsies of an ALS patient with severe clinical deterioration, suggesting that skeletal muscle may be directly affected by C9ORF72 deficiency [37].

Given the critical role of C9ORF72 in maintaining proteostasis and mitochondrial function, this study aimed to investigate the effects of its deficiency on skeletal muscle morphology and biochemical pathways. Specifically, we analyzed the muscle fiber composition and ultrastructural morphology of the gastrocnemius caput medialis (GCM), a muscle mainly formed by fast-fatigable motor units that are more prone to degeneration, in C9ORF72 knockout mice (C9^−/−^) compared to age-matched wild-type (C9^+/+^) mice. Additionally, we examined whether C9ORF72 deficiency disrupts proteostasis and mitochondrial quality control processes in skeletal muscle. These findings aim to provide a foundation for future studies exploring the interaction between neuronal and muscular pathways in ALS caused by C9ORF72 mutations and to identify novel therapeutic targets and potential disease biomarkers.

## 2. Methods

### 2.1. Generation of C9^−/−^ Mice

The constitutive knockout mouse models for the C9orf72 gene (C9^−/−^) were generated according to the protocol described previously [39]. Briefly, a floxed mouse line for the conditional deletion of C9orf72 exons 3 to 4, coding for the two C9orf72 ATG start codons (GenOway S.A., Lyon, France), was cross-bred with C57BL/6 mice that constitutively and ubiquitously expressed Cre-recombinase (under CMV promoter) (Taconic Biosciences GmbH, Koeln, Germany) to generate mice deleted for exons 3 and 4, as confirmed by qualitative PCR of the DNA from tail biopsy. The mice were bred to produce homozygous C9orf72- knockout mice under the C57BL/6J genetic background. Wild-type mice on the same genetic background were used as age-matched controls. The absence of C9orf72 expression in skeletal muscle and other tissues of C9orf72 knockout mice, together with the lack of neuromuscular impairment, as assessed by forelimb and hindlimb grip endurance on an inverted grid, has been described in a separate manuscript in which we also showed that C9orf72 localizes in motor neurons, microglia, oligodendrocytes, Schwann cells, and skeletal muscle fibers (Sironi et al. J. Neuroinflammation in press) [40].

Procedures involving animals and their care that were conducted at the Mario Negri Institute for Pharmacological Research IRCCS, Milan, Italy, adhered to the institutional guidelines, that comply with national (D.lgs 26/2014; Authorization n.493/2019-PR issued on 4 July 2019, by Ministry of Health) and Mario Negri institutional regulations and policies providing internal authorization for persons conducting animal experiments (Quality Management System certificate—UNI EN ISO 9001:2008—reg. N° 6121), the NIH Guide for the Care and Use of Laboratory Animals (2011 edition), and EU directives and guidelines (EEC Council Directive 2010/63/UE). All animals were housed under specific pathogen-free (SPF) conditions at a temperature of 22 ± 1 °C, with a relative humidity of 55 ± 10%, on a 12 h light/dark cycle, four animals per cage. Food (standard pellets) and water were supplied ad libitum.

### 2.2. Behavioral Analysis

The paw grip endurance test was used to monitor the muscle force resistance of all mice as previously described [41]. The test was performed twice a week from 12 until 22 weeks and from 42 until 52 weeks of age by the same investigator blinded to the experimental groups analyzed. The mice were placed on a horizontal metallic grid which was then gently inverted. The latency to fall of each mouse was recorded. The test ended after 90 s. If the mouse fell before 90 sec the test was repeated up to three times and the best performance was considered for the statistical analysis.

### 2.3. Muscle Tissue Collection

Adult (22 weeks old) C9^−/−^ female mice and age-matched female controls (C9^+/+^) (N = 6 per group) were deeply anesthetized with ketamine hydrochloride (150 mg/kg) and medetomidine (2 mg/kg). For immunohistochemical, biochemical, and biomolecular analyses, mice were perfused intracardially with phosphate-buffered saline (PBS) 0.1 M pH = 7.4 to remove the blood after anesthesia. The GCM muscles were rapidly dissected out, frozen in cooled isopentane and stored at −80 °C until required. For light and transmission electron microscopy (TEM), GCM, Extensor Digitorum Longus (EDL), and Tibialis Anterior (TA), muscles were dissected and fixed by 4 h immersion in aldehydic solution (4% paraformaldehyde +0.5% glutaraldehyde in 0.1M PBS). Tissues were rinsed in 0.1 M PBS and stored at 4 °C in the same buffer containing 0.4% sodium azide until further processing.

### 2.4. Morphometric Analysis of Muscle

For immunohistochemistry analyses GCM muscles were embedded in OCT mounting medium (VWR) and cut, using a cryostat (Leica Microsystem, Vienna, Austria), in transverse serial sections of 10 µm that were collected on poly-lysine objective slides (VWR International, Radnor, PA, USA).

To evaluate the cross-sectional area (CSA), the sections were fixed in cold acetone solution for 10 min, air dried and washed, and stained with Wheat Germ Agglutinin, Alexa Fluor™ 488 conjugate (1:500; Thermo Fisher, Waltham, MA, USA) and Hoechst (1:1000; Roche, Basel, Switzerland). For the muscle fiber composition, sections were fixed in acetone for 10 min, incubated in a blocking solution composed of 10% normal goat serum (NGS) and 0.1% Triton X-100 in PBS for 1 h at room temperature (RT), and then incubated overnight (O/N) at 4 °C with the primary antibody. To determine the fiber type, the sections were incubated with MyHC type I (BA-D5, 1:10; DSHB, Iowa City, IA, USA), MyHC type IIa (SC-71, 1:17; DSHB, Iowa City, IA, USA), MyHC type IIb (BF-F3, 1:9; DSHB, Iowa City, IA, USA), Rabbit anti-Laminin (1:100, L9393; Sigma-Aldrich, St. Louis, MO, USA) primary antibodies and respective secondary antibodies, anti-MIgG2b Alexa-flour 564 (A21144, 1:500) (Invitrogen, Waltham, MA, USA), anti-MIgG1 Alexa-flour 488 (A21121, 1:500) (Invitrogen, Waltham, MA, USA), anti-MIgM Alexa-flour 647 (A21046, 1:500; Invitrogen, Waltham, MA, USA), and anti-Rabbit Alexa Fluor 405 (ab175649, 1:500; Abcam, Cambridge, UK). Images were acquired with a sequential scanning mode by an A1 Nikon confocal running NIS-Elements at 20× magnification, and muscle sections were analyzed with the “MuscleJ” plug-in of Fiji software (version 1.54p) as previously described [41].

### 2.5. Real-Time PCR

Total RNA from GCM muscles was extracted using the Trizol method (Invitrogen, Waltham, MA, USA) and purified with PureLink RNA columns (Life Technologies, Carlsbad, CA, USA). RNA samples were treated with DNase I and reverse transcription was performed with the HighCapacity cDNA Reverse Transcription Kit (Life Technologies, Carlsbad, CA, USA). Real-time PCR was performed using the Taq Man Gene expression assay (Applied Biosystems, Waltham, MA, USA) following the manufacturer’s instructions, on cDNA specimens in duplicate, using the 1x Universal PCR master mix (Life technologies, Carlsbad, CA, USA) and 1x mix containing specific receptor probes for the C9orf72 gene (Mm01216829 m1; Life technologies, Carlsbad, CA, USA), Atrogin1 gene (Mm00499523_m1; Life technologies, Carlsbad, CA, USA), and Murf1 (Mm01185221_m1; Life technologies, Carlsbad, CA, USA). Relative quantification was calculated from the ratio between the cycle number (Ct) at which the signal crossed a threshold set within the logarithmic phase of the given gene and that of the reference β-actin gene (Mm02619580 g1; Life technologies, Carlsbad, CA, USA). Mean values of the duplicate results for each animal were used as individual data for 2^−ΔΔCt^ statistical analysis.

### 2.6. Western Blots

The GCM muscles were homogenized with a Teflon potter in ice-cold RIPA buffer (Abcam, Cambridge, UK) with protease and phosphatase inhibitor cocktail (Roche), then sonicated, shaken for 30 min at 4 °C, and centrifuged at 13,000 rcf for 20 min at RT. The supernatant was collected and stored at −80 °C until use.

Protein extracts were quantified using the PierceTM BCA Protein assay kit (Thermo Fisher Scientific, Waltham, MA, USA) with bovine serum albumin (BSA) as the standard. The absorbance of the solution was read at 562 nm wavelength using the Infinite^®^200 multimode reader (Tecan, Männedorf, Switzerland). A simple linear regression analysis of the BSA curve was performed to which the absorbance of samples was interpolated to estimate the protein concentration of samples.

Equal amounts of total protein homogenates were loaded on polyacrylamide gels and electroblotted onto a PVDF membrane (Millipore, Billerica, MA, USA). Membranes were incubated in blocking buffer composed of 5% BSA, dissolved in 0.1% Tween20 in Tris-buffered saline pH 7.4 (TBS-T) solution for 1 h and then probed O/N at 4 °C with the primary antibody diluted in 3% BSA in TBS-T: mouse anti-BECLIN1 (1:100, Santa Cruz, Dallas, TX, USA), mouse anti-p62 (1:500, Abcam, Cambridge, UK), rabbit anti-LC3B (1:500, Genetex, Irvine, CA, USA), rabbit anti-PGC1α (1:1000, Abcam, Cambridge, UK), rabbit anti-PARKIN (1:1000, Invitrogen, Waltham, MA, USA), mouse anti-PINK1 (1:500, Santa Cruz, Dallas, TX, USA), rabbit anti-TFEB (1:1000, Cell Signaling, Danvers, MA, USA), and mouse anti-TOM20 (1:1000, BD Transduction Laboratories, Franklin Lakes, NJ, USA). Membranes were then incubated with the opportunely diluted HRP-conjugated secondary antibody (Santa Cruz, Dallas, TX, USA) for 1 h at RT. Immunoreactivity was visualized with Immobilon Forte Western HRP substrate (Millipore, Billerica, MA, USA) at ChemiDoc XRS (Biorad, Hercules, CA, USA). The immunoreactivity was normalized to the total amount of protein loaded, as determined by Ponceau staining. The optical density of the blots was measured with Image Lab 6.1 software (BioRad, Hercules, CA, USA) and normalized to the total amount of protein loaded [42].

### 2.7. Transmission Electron Microscopy (TEM)

After dissecting small bundles of muscle from tendon to tendon, they were cut orthogonally into approximately 1 mm^3^ pieces, and processed as previously described [43]. Briefly, samples were fixed with 2% glutaraldehyde in 0.1 M cacodylate buffer (pH 7.4). After rinsing, specimens were post-fixed with reduced osmium tetroxide (1% OsO_4_ + 1% K_3_Fe(CN)_6_ in 0.1 M cacodylate buffer), rinsed again, *en-bloc* stained with X-solution (homemade contrast agent) [44], dehydrated, and embedded in epoxy resin that was backed for 48 h at 60 °C till complete resin polymerization. Samples were cut for LM and TEM analysis using an ultramicrotome (UC7, Leica Microsystem, Vienna, Austria) equipped with a 45° diamond knife (DiATOME, Nidau, Switzerland). Sections for LM (500 nm thick) were placed on glass slides, stained with 0.1% methylene blue + 0.1% toluidine blue in 240 mM phosphate buffer (pH 7.4) and imaged with an optical microscope (DM750, Leica Microsystem, Vienna, Austria), equipped with an ICC50HD (Leica Microsystem, Vienna, Austria) digital camera. Sections for TEM characterization (90 nm thick) were collected on copper grids (G300Cu—EMS, Hatfield, PA, USA), and analyzed using a Zeiss Libra 120 Plus transmission electron microscope (Carl Zeiss, Oberkochen, Germany), operating at 120 kV, equipped with an in-column omega filter for energy-filtered imaging, and a bottom mounted 16-bit CCD camera 2 k × 2 k (TRS).

### 2.8. Sample Size and Statistical Analysis

Statistical analyses were performed using GraphPad Prism version 10 (GraphPad Software, San Diego, CA, USA). Values are reported as mean ± SEM.

The sample size for biochemical and behavioral experiments was estimated through a power analysis performed with G*Power Version 3.1.9.2. using a two-tailed Student’s *t*-test with α = 0.05 and β = 0.8. Animals were excluded from analysis based on predefined criteria, including abnormal behavior or death unrelated to the experimental procedure or genotype, or technical issues during sample collection.

We evaluated the normality of each data distribution with the Shapiro–Wilk normality test, then, we used the *t*-test to check the difference between the experimental groups. For the evaluation of CSA and fiber type composition we used two-way ANOVA followed by Fisher’s LSD test.

## 3. Results

### 3.1. Loss of C9orf72 Reduces the Classes and Size of Muscle Fibers in GCM

**Figure 1:**
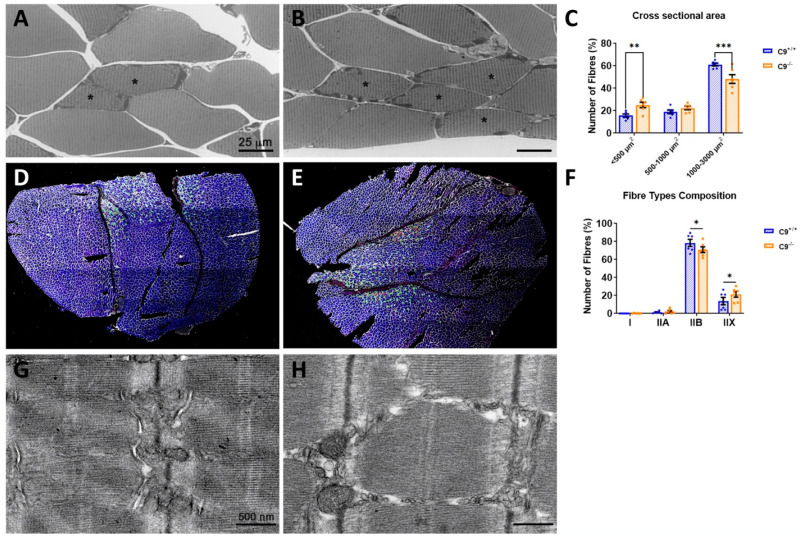
Loss of C9orf72 induces muscle atrophy and alterations in the muscle fiber composition. Representative optical images of the GCM muscle in C9^+/+^ (**A**) and C9^−/−^ (**B**) mice and myofibers mean cross-sectional area (CSA) distribution (**C**) in both experimental groups. Representative images of coronal sections of the GCM muscle in C9+/+ (**D**) and C9^−/−^ (**E**) labeled with laminin (white), myosin heavy chain (MyHC) type I (red), IIA (green), IIX (black), and IIB (blue). Analysis of the fiber type composition is shown in (**F**). Type IIX fibers correspond to unlabeled fibers. Representative electron micrographs of C9^+/+^ (**G**) and C9^−/−^ (**H**) GCM muscles, respectively. * = slow (S) motor units. Data are expressed as mean± SEM of six mice per group and examined by two-way ANOVA followed by Fisher’s LSD test. * *p* < 0.05, ** *p* < 0.01, *** *p* < 0.001.

The analysis of the overall diameter (diam) of myofibers in the GCM (Figure 1A,B) revealed a significant reduction in C9^−/−^ mice compared to C9^+/+^ controls (C9^+/+^: 35.8 ± 9.7 µm, n = 272; C9^−/−^: 31.2 ± 8.3 µm, n = 266; mean ± S.E.M.; *p* < 0.0001). A similar reduction was found in the EDL muscle, whereas the TA muscle exhibited a non-significant trend (Supplementary Figure S1).

To further investigate the reduction in GCM fiber diameter, we performed the segmentation and quantification of the CSA of the GCM fibers. This analysis showed a significant 19.5% decrease in the proportion of large fibers (1000–3000 µm^2^) and a compensatory 66% increase in small size fibers (<500 µm^2^) in C9^−/−^ mice compared to C9^+/+^ controls (Figure 1C). Next, we assessed the myofiber type composition by immunostaining for myosin type I, IIA, IIX, and IIB. Consistent with previous studies [41], we found that the GCM in mice primarily consists of fast-twitch glycolytic fibers (type II), with type IIB and IIX being the most prevalent, whereas slow-type oxidative fibers (type I red muscle) are much less represented. Interestingly, in C9^−/−^ mice, the proportion of type IIB fibers is reduced, while type IIX is increased, with no significant difference observed in type I fibers (Figure 1D–F).

Despite the reduction in myofiber diameter, the overall weight of the GCM in adult C9^−/−^ mice remained comparable to that of C9^+/+^ controls (C9^+/+^ 125.7 ± 3.8 mg, n = 3 vs. C9^−/−^, 119.9 ± 1.8 mg, n = 8; mean ± S.E.M, NS). However, a modest but significant decrease in GCM weight was observed in C9^−/−^ mice at 1 year of age (C9^+/+^ 138.6 ± 3.6 mg, n = 5 vs. C9^−/−^118.9 ± 4.8 mg, n = 8; *p* < 0.01). The body weight of C9^−/−^ mice was also significantly lower than that of C9^+/+^ mice at this age (mean ± S.D. 27.7 g ± 3.0 n = 9 vs. 31.7 g± 2.3 n = 5; *p* < 0.01, Student’s *t* test). However, no impairments were observed in the paw grip endurance test in C9^−/−^ mice, indicating the absence of functional neuromuscular defects.

### 3.2. Loss of C9orf72 Alters the Protein Degradation Pathways in GCM

**Figure 2:**
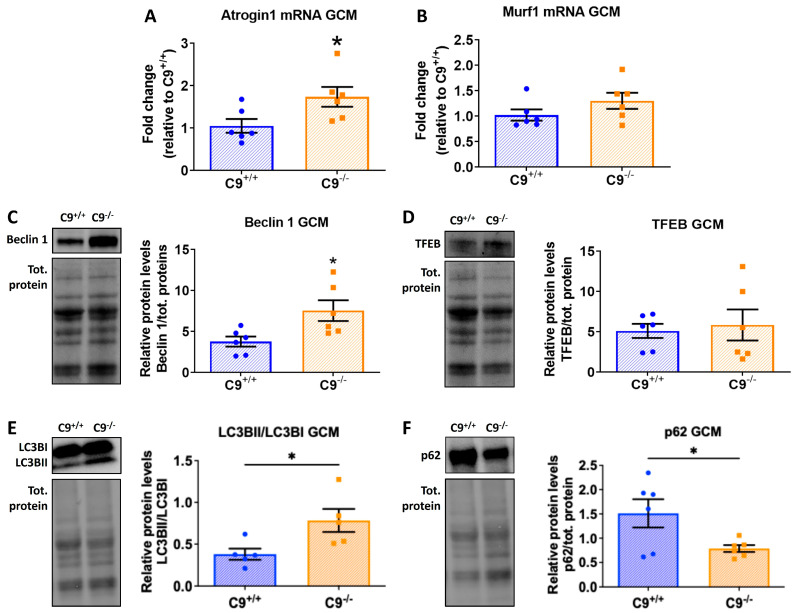
C9orf72 deletion alters proteolysis systems in the GCM of mice. (**A**,**B**) Quantitative analysis of mRNA for Atrogin 1 and MURF1, normalized on beta-actin transcript, measured by RT-PCR. (**C**–**F**) Representative immunoblot of Beclin, TFEB, LC3BII/LC3BI, and p62, with relative quantification performed on GCM lysates from C9^−/−^ mice and control C9^+/+^. The immunoreactivity for each antibody was normalized to the total amount of protein loaded, as determined by Ponceau staining. All data are expressed as mean ± SEM (n = 6 per experimental group) and analyzed by Student’s *t*-test. * *p* < 0.05.

To investigate whether reduced muscle fiber size in the GCM of C9^−/−^ mice stems from altered proteolysis, we analyzed key components of the ubiquitin–proteasome and autophagy–lysosome systems, comparing their expression level to those in C9^+/+^ mice. We assessed transcript levels of muscle-specific E3 ubiquitin ligases, Atrogin-1 (MAFbx) and MuRF1, which govern proteasomal substrate specificity. Atrogin-1 mRNA levels were significantly elevated by approximately 50% in C9^−/−^ mice compared to controls (Figure 2A), while MuRF1 showed no significant difference (Figure 2B), indicating selective activation of the proteasome system.

To assess autophagy activity, we examined Beclin 1 and TFEB levels, two master regulators of autophagy by immunoblotting. Beclin 1, which initiates autophagosome formation, was markedly increased in C9^−/−^ mice (Figure 2C), while TFEB, a transcriptional regulator of lysosomal biogenesis, showed no significant change (Figure 2D). These findings suggest an enhanced autophagy initiation that is independent of TFEB-mediated transcriptional changes.

To further understand autophagic dynamics, we evaluated the LC3BII/I ratio and the levels of receptor sequestosome 1 (SQSTM1, p62), markers of autophagy flux. The LC3BII/I ratio was significantly elevated (Figure 2E), suggesting an increased autophagosome formation and maturation. Interestingly, sporadic phagosomes and multilamellar structures which are both characteristic of autophagy processes were observed with TEM analysis only in the GCM of C9^−/−^ mice (Supplementary Figure S2). In contrast, p62 levels were reduced in C9^−/−^ mice (Figure 2F), indicating an enhanced autophagic degradation of ubiquitinated proteins. Overall, these changes suggest a functionally enhanced autophagy pathway, with active initiation, autophagosome formation, and cargo degradation, independent of TFEB transcriptional upregulation.

### 3.3. Loss of C9orf72 Alters Mitochondrial Pools and Impairs Mitophagy in GCM

**Figure 3:**
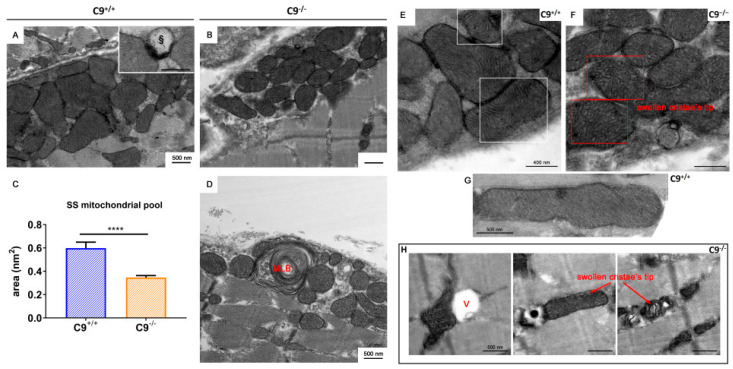
Subsarcolemmal (SS) and intermyofibrillar (IMF) mitochondrial pool. Representative electron micrographs of subsarcolemmal mitochondrial pools in WT (**A**) and C9^−/−^ (**B**) gastrocnemius muscles. § = lysosome. (**C**) Comparison of the SS mitochondrial area in WT and C9^−/−^ muscles *t*-test statistical relevance **** *p* < 0.0001. (**D**) Multilamellar body (MLB) within the SS pool of C9^−/−^ muscles. (**E**,**F**) WT and C9^−/−^ SS mitochondria, respectively. § = lysosome. (**G**) A representative healthy WT mitochondrion. (**H**) Altered IMF mitochondria observed in C9^−/−^ muscles, characterized by the alteration of cristae distribution, vacuolization processes (V), and swelling of the inner mitochondrial chamber.

Using electron microscopy, we found differences in the structure and distribution of the mitochondrial pool in the GCM of C9^−/−^ mice compared to age-matched controls. Notably, in the GCM of C9^−/−^ mice, we observed altered subsarcolemmal (SS) mitochondria, often interspersed among healthy ones (Figure 3A,B). SS mitochondria dimensions appear more uniformly distributed in C9^−/−^ mice compared to those in the GCM of wild-type mice. The average area of SS mitochondria was reduced in C9^−/−^ muscle fibers compared to age- matched C9^+/+^ (C9^+/+^: 0.60 ± 0.65 µm^2^, n = 158; C9^−/−^ 0.35 ± 0.19 µm^2^, n = 131; *p* < 0.0001) (Figure 3C). Moreover, in the GCM of C9^−/−^ mice, the presence of multilamellar bodies (MLB in Figure 3D) in the subsarcolemmal region suggests a lodged mechanism of delivery of damaged mitochondria to the lysosomal pathway. Also, in the GCM of C9^−/−^ mice, SS mitochondria appeared with flattened and disorganized *cristae* (Figure 3D), and paler matrix (Figure 3F) compared to C9^+/+^ (Figure 3E). In contrast, the analysis of the intermyofibrillar (IMF) mitochondrial pool revealed that their number, distribution, and size was comparable between the two groups. However, while the ultrastructure of C9^−/−^ IMF mitochondria was generally similar to the C9^+/+^ one, occasional vacuolization of the outer membrane was observed in C9^−/−^ mice (§ in Figure 3H).

**Figure 4:**
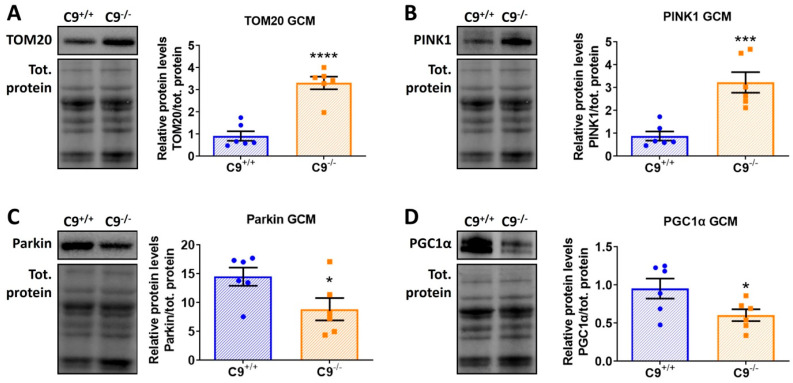
C9orf72 deletion alters mitophagy in the GCM of mice. (**A**–**D**) Representative immunoblot of TOM20 (**A**), PINK1, (**B**) Parkin (**C**), and PGC1a (**D**), and relative quantification performed on GCM lysates from C9^−/−^ mice and control C9^+/+^. All data are normalized on total amount of protein loaded, as determined by Ponceau staining and expressed as mean ± SEM (n = 6 per experimental group), and analyzed by Student’s *t*-test. * *p* < 0.05, *** *p* < 0.001, **** *p* < 0.0001.

The presence of some non-degraded altered mitochondria within lytic organelles in the GCM of C9^−/−^ mice (Figure 3D, inset square) prompted us to investigate mitophagy, the quality control mechanism responsible for removing damaged or dysfunctional mitochondria. The levels of translocase of the outer membrane 20 (TOM20), a marker for mitochondrial mass and integrity, and PTEN-induced kinase 1 (PINK1), a key regulator of mitochondrial quality control, were significantly elevated in C9^−/−^ mice (Figure 4A,B). In contrast, Parkin, an E3 ubiquitin ligase recruited by PINK1 to tag damaged mitochondria for autophagic degradation, was decreased in C9^−/−^ mice (Figure 4C), suggesting an impairment of mitophagy.

Since an increase in TOM20 could stems for an increased mitochondrial biogenesis, we analyzed the levels of peroxisome proliferator-activated receptor gamma coactivator 1 alpha (PGC1α), the master regulator of mitochondrial biogenesis. PGC1α was reduced in the GCM of C9^−/−^ mice compared to those of age-matched controls (Figure 4D), suggesting that the accumulation of TOM20 is likely due to the accumulation of damaged mitochondria in response to a defective mitophagy, rather than to increased biogenesis.

Mitochondrial alterations were also noted at the neuromuscular junction (NMJ) of C9^−/−^ mice, where resident junctional mitochondria appeared smaller, with a paler matrix and disorganized cristae (Supplementary Figure S3A,B). The structure of primary and secondary folds as well as the mitochondria distribution was not altered in C9^−/−^ mice compared to their age-matched controls.

However, in a few cases, observed only in GCM of C9^−/−^ mice, we noted altered junctions characterized by a flattened primary fold and swollen secondary ones. In these NMJs, we observed the presence of lytic organelles and densely packaged synaptic vesicles, which were not present in C9^+/+^ mice (Supplementary Figure S3C).

## 4. Discussion

The C9orf72 mutation is the most common genetic cause of ALS, linked to both gain- and loss-of-function mechanisms. However, the precise physiological role of C9orf72 across the various tissues implicated in ALS remains poorly understood. This study provides the first in-depth examination of C9orf72 function in skeletal muscle, a tissue increasingly recognized as a possible primary target in the pathogenesis of ALS [32].

Our findings indicate that loss of C9orf72 alters muscle morphology, disrupts protein degradation pathways, and impairs mitochondrial dynamics, potentially contributing to an ALS phenotype when combined with the gain-of-function effects from DPR toxicity [25]. Notably, reduced C9orf72 protein expression has been observed in muscle fibers from an ALS patient at autopsy, accompanied by p62- and ubiquitin-positive cytoplasmic aggregates [37]. However, iPSC-derived skeletal myocytes from C9ALS patients exhibited pathological features, including mitochondrial dysfunction, elevated oxidative stress, and abnormal proteostasis, despite normal C9ORF72 expression level [45].

Although our C9orf72 knockout mice displayed a partial skeletal muscle denervation [40] and shift toward smaller muscle fibers (the present study), we did not observe neuromuscular impairment, consistent with prior reports describing mild or absent phenotypes in C9orf72 loss-of-function models [19,20,21,22,23]. Together, these observations suggest that both loss- and gain-of-function mechanisms may converge in skeletal muscle to drive disease pathology in C9ALS, highlighting skeletal muscle as a critical and previously underappreciated contributor to ALS progression.

### 4.1. Muscle Morphology and Fiber-Type Remodeling

We demonstrated that the absence of C9orf72 leads to a significant reduction in the CSA of GCM muscle fibers in adult mice. Notably, there was a selective decrease in large, metabolically demanding fast-twitch glycolytic type IIB fibers, whereas smaller fibers, in particular the type IIX, were expanded compared to those of C9^+/+^ controls. This shift likely reflects an adaptive response to reduced innervation and altered energy homeostasis. The observed remodeling may result from the significant decrease in the PGC-1α expression and mitochondrial biogenesis, leading to reduced oxidative capacity [46]. Despite these structural abnormalities, overall skeletal muscle mass was preserved in adult mice, consistent with the absence of an overt phenotype. However, in one year old mice (middle aged), there was a modest but significant decline of GCM weight compared to age- matched controls. Importantly, this reduction did not impair muscular endurance, as assessed by the grip strength test. Collectively, these findings suggest a compensatory remodeling of muscle fiber types in response to the C9orf72 deficiency which may sustain muscle function over time. A mild reduction in muscle mass does not necessarily impair grip strength endurance in absence of motor neuron dysfunction [41,47], and although our C9^−/−^ mice exhibit partial muscle denervation, they show no evidence of motor neuron loss [40].

### 4.2. Proteostasis

Mechanistically, we found that C9orf72 loss disrupts proteostasis in skeletal muscle. Specifically, we observed upregulation of the E3 ubiquitin ligase Atrogin-1, which likely accelerates protein turnover and contributes to atrophy in glycolytic fibers, such as type II, which are more susceptible to catabolic signals. The selective activation of Atrogin-1, but not MuRF1, is consistent with evidence that these atrogenes are regulated by distinct pathways. For example, Nishimura and colleagues [48] reported that Atrogin-1, but not MuRF1, is upregulated when the mTOR-S6K1 pathway is inhibited. This aligns with findings by Ugolino and coworkers [29], who demonstrated that C9orf72 loss inhibits mTOR-S6K1 signaling in human cell lines and mouse embryonic fibroblasts. Moreover, Atrogin-1 has been tightly linked to protein breakdown [49]. Therefore, we hypothesize that the reduction in the large muscle fibers of GCM in C9^−/−^ mice may result from Atrogin-1 activation, driven by mTORC inhibition in the absence of C9orf72.

### 4.3. Autophagy

In addition to proteasomal dysregulation, we found that autophagy pathways were altered in the GCM of C9^−/−^ mice. Consistent with previous reports in experimental C9orf 72-depleted models [28,29], we observed a significant increase in the LC3II/I ratio, along with reduced p62/SQSTM1 levels, indicative of enhanced autophagy-mediated protein degradation. However, TFEB was not upregulated, suggesting that the regulation of autophagy may differ across cell types [29]. Interestingly, the activation of autophagy in skeletal muscle in C9^−/−^ mice contrasts with the effect of C9orf72 deficiency observed in the nervous system. Specifically, while cortical levels of LC3B-I, LC3B-II, and p62 remain unchanged in C9ORF72 knockout mice, loss of endogenous C9ORF72 suppressed the DPR-induced autophagy activation [25]. Furthermore, studies in neuronal cell lines have shown that C9orf72 knockdown impairs serum starvation-induced autophagy as evidenced by reduced LC3B-I to LC3B-II conversion, impaired p62 degradation, and decreased formation of LC3-positive autophagosomes [30]. Consistent with this, C9orf72 loss exacerbates TDP-43 pathology and neurodegeneration by hindering autophagic clearance of cytoplasmic aggregates both in vitro and in vivo in TDP25-expressing mice [50]. Together, these findings highlight a tissue-specific divergence in the autophagic response to C9orf72 loss, with distinct regulatory mechanisms in skeletal muscle and neurons that contribute to pathological outcomes such as muscle wasting and protein aggregation in the presence of DPR or other ALS-linked mutations. Although activation of the autophagic pathway has been proposed as a therapeutic strategy to mitigate the toxic accumulation of insoluble proteins in the CNS [51], its stimulation in skeletal muscle may be detrimental in C9orf72 ALS.

### 4.4. Mitophagy and Mitochondrial Function

Our study also highlights a potential role for C9orf72 in mitochondrial homeostasis. C9orf72 is known to regulate mitochondrial function by interacting with the prohibitin complex, which controls cristae morphogenesis and mitochondrial integrity [29]. In C9^−/−^ mice, we observed abnormalities in subsarcolemmal mitochondria of GCM fibers, including disrupted cristae and uniformity in size. Under normal condition, PINK1 ubiquitinates the defective mitochondria to be rapidly degraded by E3 ubiquitin ligase Parkin [52]. In the absence of C9orf72, defective recruitment of Parkin, likely due to the reduced protein levels, may lead to an accumulation of damaged mitochondria. This aligns with the increased levels of PNK1 and TOM20, suggesting a failure to clear damaged mitochondria. This scenario points to an incomplete or impaired mitophagy process, where mitochondria are recognized as damaged by PINK1 but cannot progress to the degradation phase due to reduced Parkin. The direct effect of C9orf72 loss on Parkin levels remains unclear. C9orf72 deficiency may indirectly regulate Parkin abundance or activity by influencing PARK2 transcription, modulating Parkin stability through effects on mitochondrial integrity, or impairing its recruitment to damaged mitochondria. It is also possible that sustained activation of autophagy and proteasome such as those occurring in C9orf72 deficiency or following denervation, leads to a repression and degradation of PGC-1α, a master regulator of mitochondrial biogenesis that we found to be reduced in the skeletal muscle of C9^−/−^ mice; this may establish a self-reinforcing pathological loop that may lead to a secondary loss of Parkin and mitophagy efficiency. In the absence of Parkin, accumulation of its substrate Parkin Interacting Substrate (PARIS), a transcriptional repressor of PGC1α, further suppresses PGC1α expression, as demonstrated in neurons [53] and skeletal muscle [54]. Consistent with this, skeletal muscle from Parkin knockout mice displays dysfunctional mitochondria and reduced myofiber CSA [55].

Taken together, these findings reveal that loss of C9orf72 disrupts multiple muscle homeostatic pathways, extending beyond its well-established roles in the brain [29,39] and motor neurons [11]. Table 1 summarizes the effects of C9orf72 loss on proteostasis and mitochondrial integrity in skeletal muscle.

Despite morphological changes, proteostasis dysfunction, and mitochondrial abnormalities, muscle force remains preserved up to middle age, likely due to the functional reserve of skeletal muscle and compensatory mechanisms such as motor unit remodeling and fiber-type switching. Similar resilience has been observed in ALS mouse models expressing SOD1G93A, where muscle force is maintained despite significant proteostasis dysfunction and muscle mass loss [41].

## 5. Conclusions

In conclusion, this study uncovers a previously unrecognized role of C9orf72 in skeletal muscle maintenance. Loss of C9orf72 function disrupts muscle morphology, proteostasis, and mitochondrial integrity, with potential implications for ALS pathogenesis alongside C9orf72 HRE–linked gain-of-function mutations. In contrast to its loss in neurons, which exacerbates TDP-43 pathology by impairing autophagic clearance, our findings reveal a specific activation in the autophagic response to C9orf72 deficiency in muscle, suggesting distinct tissue-specific regulatory mechanisms. These observations extend the impact of C9orf72 loss of function beyond the nervous system, highlighting a shared vulnerability of post-mitotic tissue to autophagy and mitochondrial dysfunction.

Although further investigation into neuronal and muscle-specific consequences of C9orf72 deficiency are needed, these findings highlight the importance of preserving C9orf72 levels in ALS patients undergoing treatments with C9orf72 antisense oligonucleotide (ASO) strategies [56]. Interestingly, despite these abnormalities, muscle force is preserved for a prolonged period in the C9^−/−^ mice, suggesting that skeletal muscle is able to undergo remarkable resilience through compensatory mechanisms. Identifying these mechanisms may help in the development of novel therapeutic strategies while the early detection of muscle-specific changes may aid in developing prodromal biomarkers for disease progression.

## Figures and Tables

**Table 1:** Main effects of C9orf72 loss in gastrocnemius muscle (GCM).

Parameter	Response in Mice GCM C9^−/−^ vs. C9^+/+^	Interpretation
Myofiber diameter	↓ (~13%)	Shift toward smaller muscle fibers
Fiber size distribution	↓ large fibers (−19.5%), ↑ small fibers (+66%)	
Fiber type composition	↓ Type IIB, ↑ Type IIX, Type I unchanged	Altered fiber-type balance (fast-to-intermediate)
Muscle weight	Adult: nc; 1 yr: ↓	Preserved muscle mass and function
Grip endurance	No change	
Proteasome	↑ Atrogin-1 nc MURF1	Enhanced proteasomal activity with selective E3 ligase activation
Autophagy	↑ Beclin 1 ↑ LC3B II/I ↓ p62/SQSTM1 nc TFEB	Increased autophagy initiation, autophagosome formation and autophagic flux
Mitochondria/mitophagy	↓ 40% SS mito area Disorganized cristae, pale matrix	Structural damage
	Multilamellar bodies ↑ TOM20, ↑ PINK1	Impaired clearance and accumulation of damaged mitochondria
	↓ Parkin	Blocked mitophagy execution
	↓ PGC1α	Decreased mitochondrial biogenesis
Neuromuscular junction	Flattened, swollen folds in a subset and smaller, disorganized mitochondria	Mitochondrial dysfunction and local synaptic stress

Legend: ↑/↓: Increased/decreased; nc: no change.

## Data Availability

The datasets used and/or analyzed during the current study are available from the corresponding author on reasonable request.

## Supplementary Materials

The following supporting information can be downloaded at: https://www.mdpi.com/article/10.3390/cells14221765/s1, Figure S1: Fibers size in Extensor Digitorum Longus (EDL) and Tibialis Anterior (TA) muscles. Left panel: representative LM images of EDL and TA of WT and C9^−/−^ muscle, respectively. Right panel: average fibers size in EDL and TA muscles. (EDL: WT: 37.4 ± 8.2; n = 82; C9^−/−^: 30.8 ± 6.8; n = 93; *P* < 0.0001; TA: WT: 50.2 ± 12.0; n = 37; C9^−/−^: 45.1 ± 12.7; n = 52; ns); Figure S2: Representative image of autophagic phenomenon in the GCM of C9^−/−^ mice. Sarcomere structures are not seeable instead it is possible to observe both lysosomes (L), Phagosomes (Ph) and Multilamellar structures (MLS) which are both characteristic of autophagy processes. Some not affected mitochondria (M) are still present in the altered muscular fibers (scale bar 500 nm).; Figure S3: NMJs in gastrocnemius muscle. (A,B) Representative electron micrographs of two innervated NMJ in C9^+/+^ and C9^−/−^ respectively. The structure of primary and secondary folds is not altered in C9^−/−^ compared to C9^+/+^. We did not observe any alteration of synaptic vesicles and mitochondria number and distribution. However, we found similar trend of alteration in junctional mitochondria of C9^−/−^ that we described for SS mitochondrial pool (e.g., smaller mitochondria, with paler matrix and disorganized cristae). C) The primary fold is flattened (black arrows) compared to not altered junctions but is still possible to recognize swollen secondary folds (§). The presynaptic terminal shows mitochondria (M) and regions with a dense packaging of synaptic vesicles (*).

## Abbreviations

ALS	amyotrophic lateral sclerosis
ASO	antisense oligonucleotide
C9ORF72	chromosome 9 open reading frame 72
ChAT	choline acetyltransferase
CSA	cross-sectional area
DPRs	dipeptide repeat proteins
DENN	differentially expressed in normal cells and neoplasia
EDL	Extensor Digitorum Longus
FTD	frontotemporal dementia
GCM	gastrocnemius caput medialis
HRE	hexanucleotide repeat
IMF	intermyofibrillar
MAFbx	muscle-specific E3 ubiquitin ligases
MyHC	Myosin Heavy Chain
MuRF1	Muscle RING Finger 1
mTORC1	Mammalian Target of Rapamycin Complex 1
NGS	normal goat serum
NMJ	neuromuscular junction
PARIS	Parkin Interacting Substrate
PBS	phosphate-buffered saline
PGC1	peroxisome proliferator-activated receptor gamma coactivator 1 alpha
PINK1	PTEN-induced kinase 1
RAN	repeat-associated non-ATG
SPF	specific pathogen-free
SQSTM1	p62, sequestosome 1
TA	Tibialis Anterior
TEM	transmission electron microscopy
TFEB	transcription factor EB
TOM20	translocase of the outer membrane 20
