# Supplementary material for: C9ORF72 Is Pivotal to Maintain a Proper Protein Homeostasis in Mouse Skeletal Muscle
Source: Cells. 2025 Nov 11;14(22):1765. doi: 10.3390/cells14221765 (PMC12651509; doi:10.3390/cells14221765)
Supplement: Supplementary file 1 [file cells-14-01765-s001.zip › cells-3865014-supplementary.pdf]

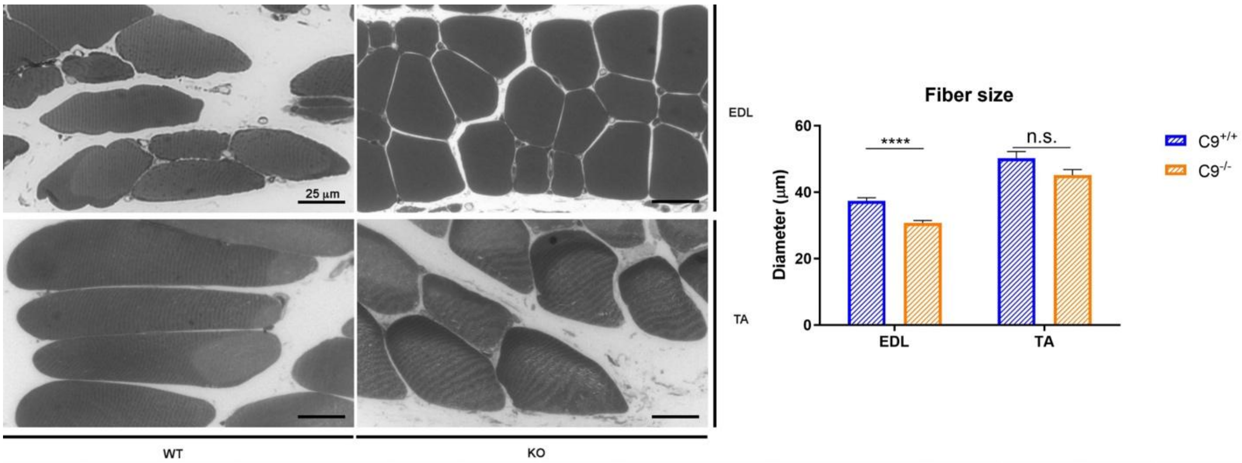

Supplementary Figure S1. Fibers size in Extensor Digitorum Longus (EDL) and Tibialis Anterior (TA) muscles. Left panel: representative LM images of EDL and TA of WT and $C9^{-/-}$ muscle, respectively. Right panel: average fibers size in EDL and TA muscles. (EDL: WT: 37.4 ± 8.2; n = 82; $C9^{-/-}$: 30.8 ± 6.8; n = 93; P < 0.0001; TA: WT: 50.2 ± 12.0; n = 37; $C9^{-/-}$: 45.1 ± 12.7; n = 52; ns)

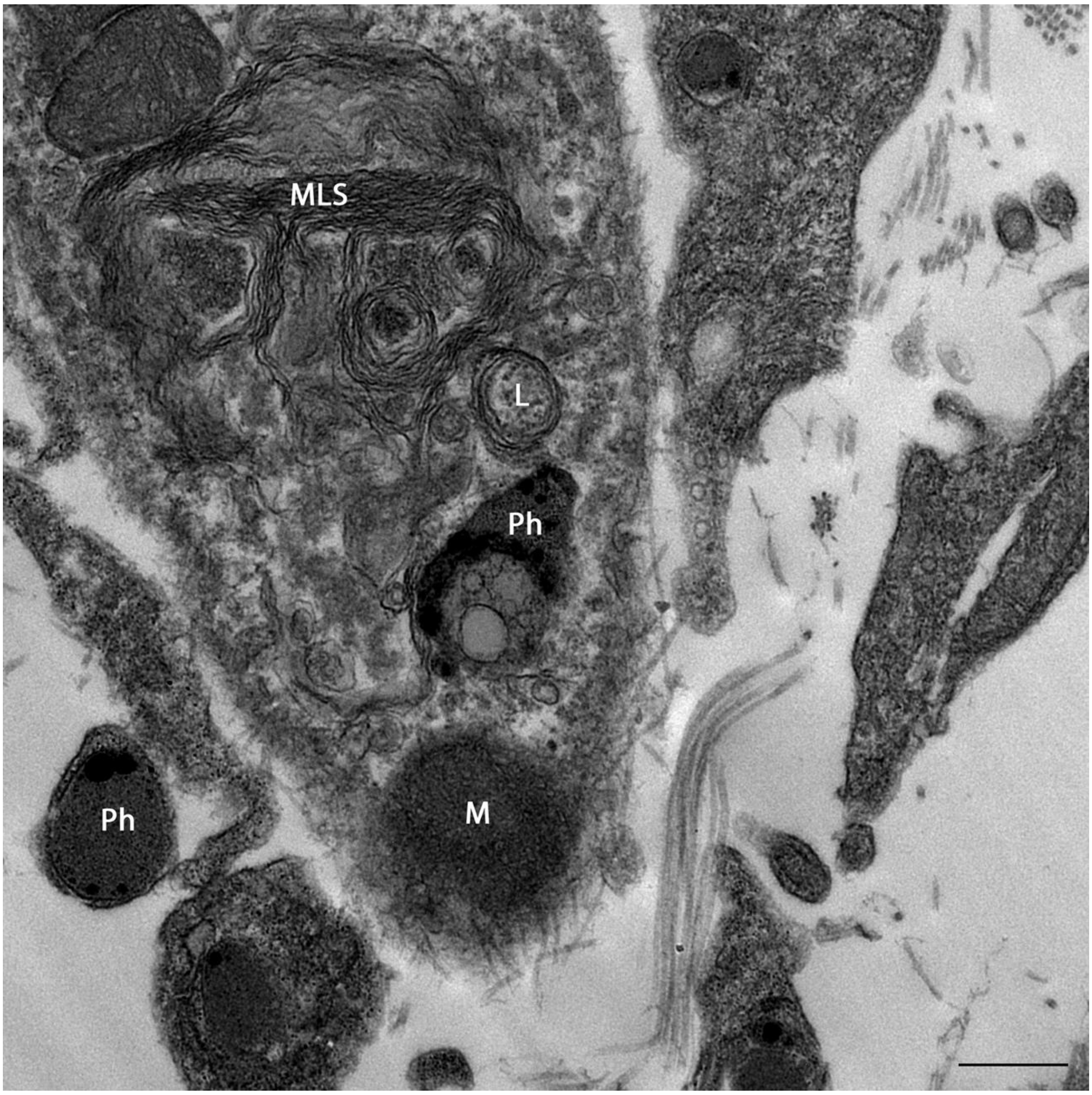

Supplementary Figure S2. Representative image of autophagic phenomenon in the GCM of C9-/- mice. Sarcomere structures are not seeable instead it is possible to observe both lysosomes (L), Phagosomes (Ph)

and Multilamellar structures (MLS) which are both characteristic of autophagy processes. Some not affected mitochondria (M) are still present in the altered muscular fibers (scale bar 500 nm).

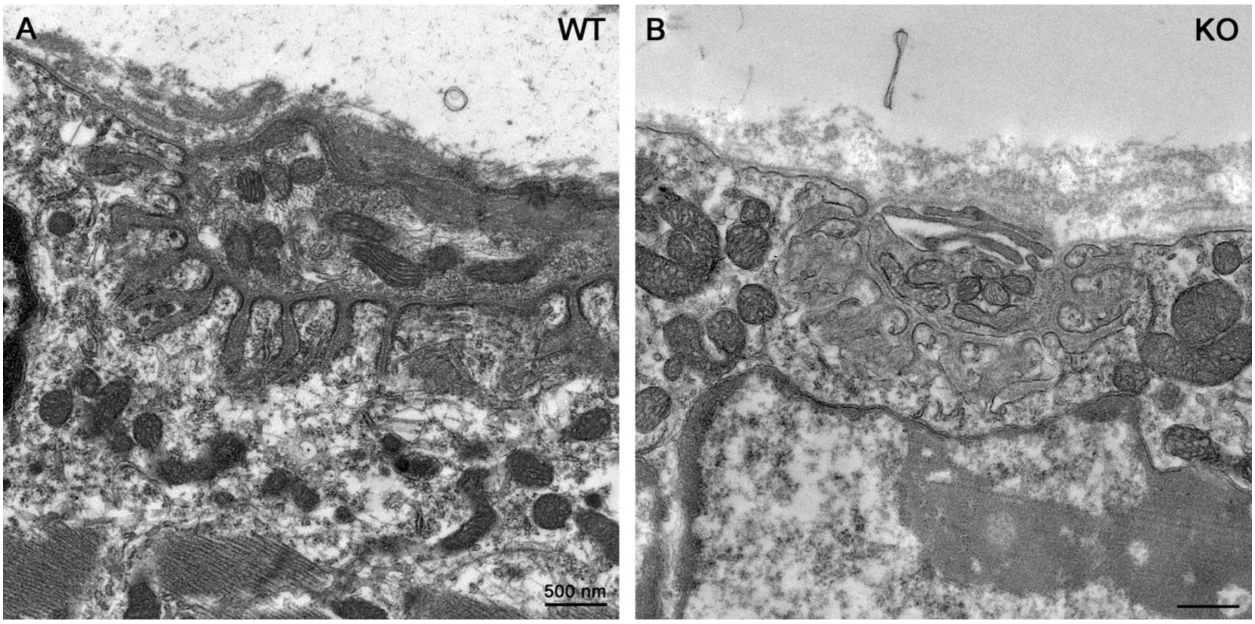

Supplementary Figure S3. NMJs in gastrocnemius muscle.

A, B) Representative electron micrographs of two innervated NMJ in $C9^{+/+}$ and $C9^{-/-}$ respectively. The structure of primary and secondary folds is not altered in $C9^{-/-}$ compared to $C9^{+/+}$. We did not observe any alteration of synaptic vesicles and mitochondria number and distribution. However, we found similar trend of alteration in junctional mitochondria of $C9^{-/-}$ that we described for SS mitochondrial pool (e.g. smaller mitochondria, with paler matrix and disorganized cristae). C) The primary fold is flattened (black arrows) compared to not altered junctions but is still possible to recognize swollen secondary folds (§). The presynaptic terminal shows mitochondria (M) and regions with a dense packaging of synaptic vesicles (*)
